# Xuebijing injection improves the respiratory function in rabbits with oleic acid-induced acute lung injury by inhibiting IL-6 expression and promoting IL-10 expression at the protein and mRNA levels

**DOI:** 10.3892/etm.2014.1949

**Published:** 2014-09-05

**Authors:** YUXIA WANG, MINGLI JI, LEI WANG, LIPING CHEN, JING LI

**Affiliations:** 1 Department of Pathophysiology, Xinxiang Medical University, Xinxiang, Henan 453003, P.R. China; 2 Department of General Surgery, The First Affiliated Hospital of Xinxiang Medical University, Weihui, Henan 453100, P.R. China; 3 Department of Physiology, Xinxiang Medical University, Xinxiang, Henan 453003, P.R. China; 4 Department of Otorhinolaryngology, The First Affiliated Hospital of Xinxiang Medical University, Weihui, Henan 453100, P.R. China

**Keywords:** Xuebijing injection, acute lung injury, oleic acid, interleukin 6, interleukin 10, myeloperoxidase

## Abstract

Xuebijing injection is a complex herbal medicine, and clinical and experimental studies have shown that it has a significant effect on acute respiratory distress syndrome and multiple organ dysfunction syndrome. However, the majority of studies regarding Xuebijing injection have focused on serum inflammatory factors, and few studies have been carried out from the perspective of the protein and mRNA expression of inflammatory cytokines. In this study, 60 healthy rabbits of mixed gender were randomly assigned to a normal control group (CG), oleic acid group (model group; MG) and oleic acid + Xuebijing injection group (treatment group; TG). Rabbits of the CG were treated with normal saline through the ear vein, rabbits of the MG were injected with oleic acid (0.4 ml/kg) and rabbits of the TG received 0.4 ml/kg oleic acid + 10 ml/kg Xuebijing injection. Blood samples were collected from the common carotid artery of all rabbits of all groups 1 h after the ear vein was injected with the corresponding reagent, and was used to measure the arterial partial pressure of oxygen (PaO_2_) and of carbon dioxide (PaCO_2_). The activity of myeloperoxidase (MPO) was tested, and the protein and mRNA expression levels of interleukin (IL)-6 and IL-10 were determined. Rabbits of the MG exhibited evident respiratory dysfunction (PaO_2_ and PaCO_2_ were low), histopathological lung damage and overactive inflammatory responses (the expression of the proinflammatory cytokine IL-6 and the anti-inflammatory cytokine IL-10 was increased at the protein and mRNA levels). Following the administration of the Xuebijing injection, the inflammatory response of the rabbits was significantly reduced. Xuebijing injection raised PaO_2_ and PaCO_2_, weakened the activity of MPO in the lung tissue, downregulated the expression of the proinflammatory cytokine IL-6 and further increased the expression of the anti-inflammatory cytokine IL-10. These results demonstrated that Xuebijing injection improved the respiratory function of rabbits with acute oleic acid-induced lung injury by inhibiting IL-6 expression and promoting IL-10 expression.

## Introduction

A previous epidemiological study has shown that acute lung injury (ALI) is common in clinical critical diseases. Despite competent supportive therapy and care having been used, the mortality rate of ALI remains high and the effectiveness remains unsatisfactory (1). ALI and its severe form, multiple organ dysfunction syndrome (MODS), remain a major public health problem. Therefore, understanding the pathogenesis of ALI/MODS and the search for novel methods to treat ALI/MODS has become currently urgent. This study focuses on the efficacy and the mechanisms of Xuebijing injection in the treatment of ALI/MODS.

An early characteristic of ALI is the activation and recruitment of neutrophils in the lungs and the inflammatory effect induced by neutrophils (2). Myeloperoxidase (MPO) is a neutrophil-specific reductase, the intracellular content of which is stable, and the number of activated neutrophils may be indirectly reflected by the MPO activity of unit lung tissue (3).

Previous studies (4–6) have shown that a variety of cytokines play key roles in the progression of ALI. Cytokines (7) that are synthesized and secreted by certain immune and non-immune cells are a type of small molecule protein with broad biological activities that are mainly involved in regulating immune response, mediating inflammation and stimulating hematopoietic function and tissue repair. Cytokines can be divided into pro- and anti-inflammatory cytokines according to their different roles in inflammatory reactions (8–10). Interleukin (IL)-6 is a potent proinflammatory cytokine, which has a crucial effect on the initiation of inflammation (11) and plays an important role in processes including host defense, acute phase response, immune response and blood reactions (12–14). Moreover, anti-inflammatory cytokines including IL-4 (15), IL-10 (16,17) and transforming growth factor-β (TGF-β) (18), are also found to be at a relatively high level in ALI.

Xuebijing injection is a herbal medicine consisting of Flos Carthami, *Salvia miltiorrhiza*, Radix Paeoniae Rubra, Chinese Angelica and *Ligusticum chuanxiong* Hort. In China, Xuebijing injection is widely used for treating acute and severe diseases including systemic inflammatory response syndrome (SIRS) and MODS (19). A previous study confirmed that the effectiveness of Xuebijing injection is associated with regulation of the expression of inflammatory cytokines at the serum level (20); however, there are limited studies concerning the regulatory effect of Xuebijing injection on the expression levels of inflammatory cytokines at the protein and mRNA levels. Thus, the current study further explored the possible mechanisms of the effect of Xuebijing injection on ALI at the protein and mRNA levels.

## Materials and methods

### Laboratory animals

Sixty healthy adult mixed gender New Zealand rabbits weighing 250–350 g were obtained from the Experimental Animal Center of Zhengzhou University (Zhengzhou, China) and used for all the experiments. They were randomly divided into three groups: the normal control group (CG; n=20), oleic acid group (model group, MG; n=20), and oleic acid + Xuebijing injection group (treatment group, TG; n=20). All rabbits were housed in separate cages under suitable temperature and humidity and had free access to water and food prior to the study. All experimental procedures were approved by the Ethics Committee of Xinxiang Medical University (Xinxiang, China). The study was performed according to the principles enunciated in the ethical principles of the Declaration of Helsinki.

### Main reagents and instruments

The main instruments used in this study include the Leica RM2235 microtome (Leica Biosystems GmbH, Nussloch, Germany), Labnet polymerase chain reaction (PCR) instrument (Shenzhen Cy-tech Biotechnology Co., Ltd., Shenzen, China), DG5033A enzyme-linked immunosorbent assay (ELISA) Analyzer (Nanjing Huadong Electronics Group Medical Equipment Co., Ltd., Nanjing, China) and the fully automatic M248 blood gas analyzer (Bayer China Ltd., Shanghai, China). The main reagents used include oleic acid (purity ≥99.0%, Sigma-Aldrich, St. Louis, MO, USA), Xuebijing injection (10 ml/vial, Tianjin Chase Sun Pharmaceutical Co., Ltd., Tianjin, China), 20% urethane (Shanghai Yunqiang Chemical Co., Ltd., Shanghai, China), IL-6 and IL-10 goat anti-rabbit polyclonal antibodies (Beijing Boosen Biological Technology Co., Ltd., Beijing, China), ELISA kits (Beijing Zhongshan Golden Bridge Biological Technology Co., Ltd., Beijing, China), MPO kits (Nanjing Jiancheng Biological Engineering Institute, Nanjing, China), TRIzol^®^ and reverse transcription (RT)-PCR kit (Baiyi Xinchen Biotechnology Co., Ltd., Beijing, China).

### Surgery

All rabbits received general anesthesia with 20% urethane (5 ml/kg) via the ear vein; the trachea and common carotid artery were isolated and intubated. Rabbits of the CG received 0.9% saline via the ear vein (0.4 ml/kg). For rabbits of the MG, the rabbit model of ALI was established according to the method of a previous study (21); rabbits were administered oleic acid (0.4 ml/kg) from the ear vein (slowly and uniformly, 0.1 ml/min). Rabbits of the TG received Xuebijing injection (10 ml/kg) immediately subsequent to injection with oleic acid by the same method as was used in the MG. When pink foamy liquid poured from the tracheal intubation of the rabbits of the MG and was accompanied by evident shallow frequency breathing, the ALI model of the MG was considered to have succeeded. Arterial blood (10 ml) was extracted from the common carotid artery of each rabbit of each group 1 h after injection of the corresponding reagent (several preliminary experiments found that it took ~1 h to establish the ALI model). Measurements of the arterial partial pressure of oxygen (PaO_2_) and carbon dioxide (PaCO_2_) in arterial blood were made using a blood gas analyzer. Later, all rabbits were sacrificed by air embolism and the lungs were removed and fixed with paraformaldehyde solution (1 ml/100 g).

### Colorimetry

MPO activity provides indirect evidence of neutrophil infiltration. MPO activity was measured according to the instructions provided by the manufacturer of the MPO kit; lung tissues were weighted accurately, 5% homogenate was prepared (weight by volume: 1:9), without centrifugation, mixed, put in a 60°C water bath for 10 min. Then, at 460 nm with a 1 cm optical path, and the use of distilled water to zero the colorimeter, the absorbance value of each tube was measured. Lung MPO activity was calculated using the following formula: MPO activity (U/g) = Optical density value of measuring tube - optical density value of control tube/11.3 × sample size (g)

### Immunohistochemistry

To evaluate IL-6 and IL-10 levels, the formalin-fixed and paraffin-embedded lung tissues were deparaffinized in xylene and rehydrated using a graded series of alcohol, quenched with 3% H_2_O_2_, blocked with 5% normal goat serum and probed with rat anti-rabbit IL-6/IL-10 antibody. Detection was with biotinylated anti-rat immunoglobulin G, followed by incubation with avidin-biotin complex and 3,3′-diaminobenzidine (DAB) substrate and hematoxylin counterstaining. According to the method described in a previous study (22), the expression levels of the two proteins were determined semi-quantitatively. Ten high-power fields were selected and the expression level was defined as follows: No positive cells, −; 0–25%, +; 25–50%, +; 50–75%, ++; >75%, +++. These experiments were performed in triplicate.

### Total RNA extraction and RT-PCR

In order to obtain the relative quantification of specific gene expression and statistical analysis compared with the CG, ~100 mg lung tissue was stored at −80°C. Total RNA was extracted by a one-step method with TRIzol^®^, and cDNA was then generated for the RT-PCR analysis, using GAPDH as an internal reference gene. The following primers (Baiyi Xinchen Biotechnology Co., Ltd.) were used: GAPDH, forward, 5′-AGTTCAACGGCACAGTCAAGG-3′ and reverse, 5′-AGACTCCACGACATACTCAGC-3′, amplified fragment length 1,622bp; IL-6, forward, 5′-CTTCCAGCCAGTTGCCTTCT-3′ and reverse, 5′-GAG AGCATTGGAAGTTGGG-3′, amplified fragment length 496 bp; IL-10, forward, 5′-CAGACCCACATGCTCCG AGA-3′ and reverse, 5′-CAAGGCTTGGCAACCCAAGTA-3′, amplified fragment length 141 bp. The gray ratio of the PCR target band and internal reference band was analyzed using Band Leader 3.0 software (Magnitec Ltd., Tel Aviv, Israel).

### Statistical analysis

Statistical analysis was performed using SPSS statistical software, version 11.0 (SPSS, Inc., Chicago, IL, USA). Measurement data are expressed as mean ± standard deviation, and were tested using the independent samples Student’s t-test. Count data were analyzed using the χ^2^ test. Spearman’s rank correlation analysis was used to analyze the correlation of IL-6 and IL-10 of rabbit lung tissue in the three groups. P<0.05 was considered to indicate a statistically significant result.

## Results

### Xuebijing injection improves the PaO_2_ and PaCO_2_ of rabbits with oleic acid-induced ALI

**Figure 1:**
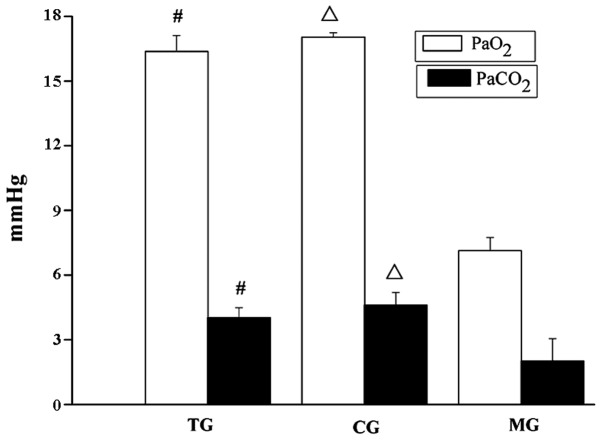
Xuebijing injection improved the PaO_2_ and PaCO_2_ of rabbits with acute lung injury induced by oleic acid. Data are presented as the mean ± standard deviation. These experiments were conducted independently in triplicate. ^#^P<0.001 and ^Δ^P<0.001 compared with the MG. TG, treatment group; CG, control group; MG, model group; PaO_2_, arterial partial pressure of oxygen; PaCO_2_, arterial partial pressure of carbon dioxide.

PaCO_2_ and PaO_2_ are used to evaluate respiratory function. PaO_2_ reduces with/without increases in PaCO_2_ during respiratory dysfunction. PaCO_2_ and PaO_2_ were measured using a blood gas analyzer. As shown in Fig. 1, PaO_2_ and PaCO_2_ in the TG were higher compared with that in the MG (P<0.001) and lower compared with those in the CG (P≥0.05) and PaO_2_ and PaCO_2_ in the MG were lower compared with those in the CG (P<0.001), which suggests that respiratory function improved when rabbits with ALI were treated with Xuebijing injection.

### Xuebijing injection decreases MPO activity in the lung tissue of rabbits with oleic acid-induced ALI

**Figure 2:**
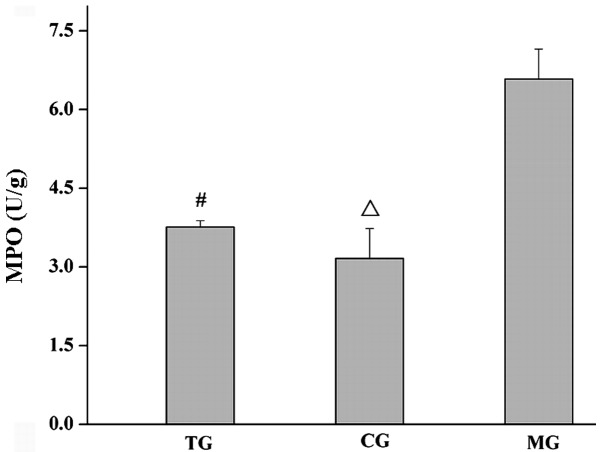
Xuebijing injection decreased MPO activity in the lung tissue of rabbits with acute lung injury induced by oleic acid. Data are presented as the mean ± standard deviation. These experiments were conducted independently in triplicate. ^#^P<0.001 and ^Δ^P<0.001 compared with the MG. TG, treatment group; CG, control group; MG, model group; MPO, myeloperoxidase.

To elucidate the underlying mechanisms by which Xuebijing injection promotes respiratory function, the MPO activity per unit of lung tissue, which is an index of neutrophil infiltration, was analyzed. It was found that the MPO activity in the lung tissue of the TG was lower compared with that of the MG (P<0.001) and higher compared with that of the CG (P≥0.05), and the MPO activity in the lung tissue of the MG was higher compared with that of the CG (P<0.001), as shown in Fig. 2.

### Xuebijing injection reduces the expression of IL-6 and increased the expression of IL-10 at the protein and mRNA levels in the lung tissue of rabbits with oleic acid-induced ALI

**Figure 3:**
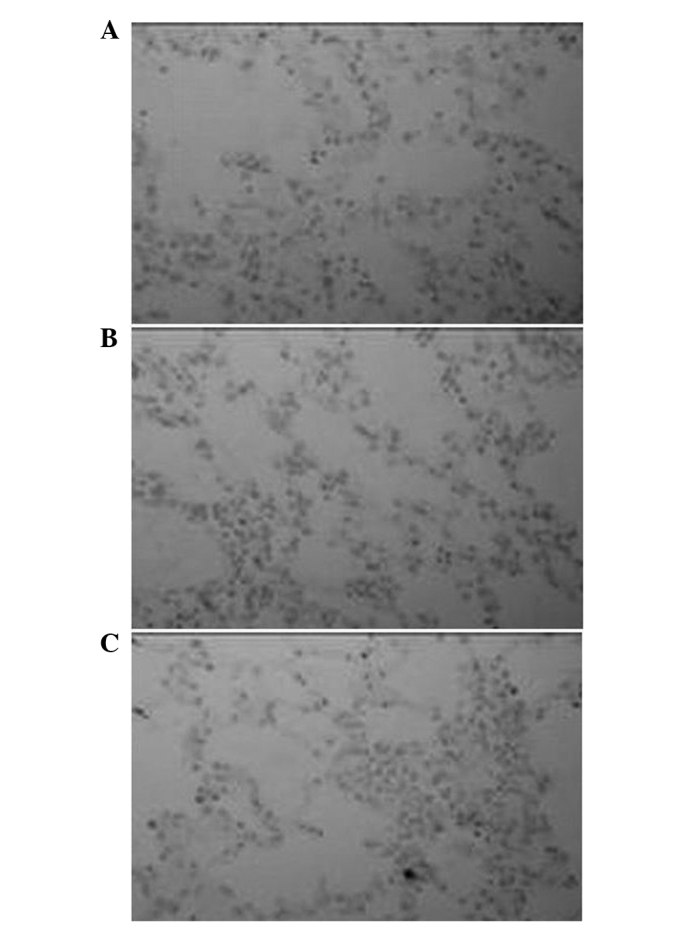
Xuebijing injection inhibited the protein expression of the proinflammatory cytokine interleukin 6. Immunohistochemically stained tissue in the (A) treatment, (B) control and (C) model groups. Magnification, ×400.

**Figure 4:**
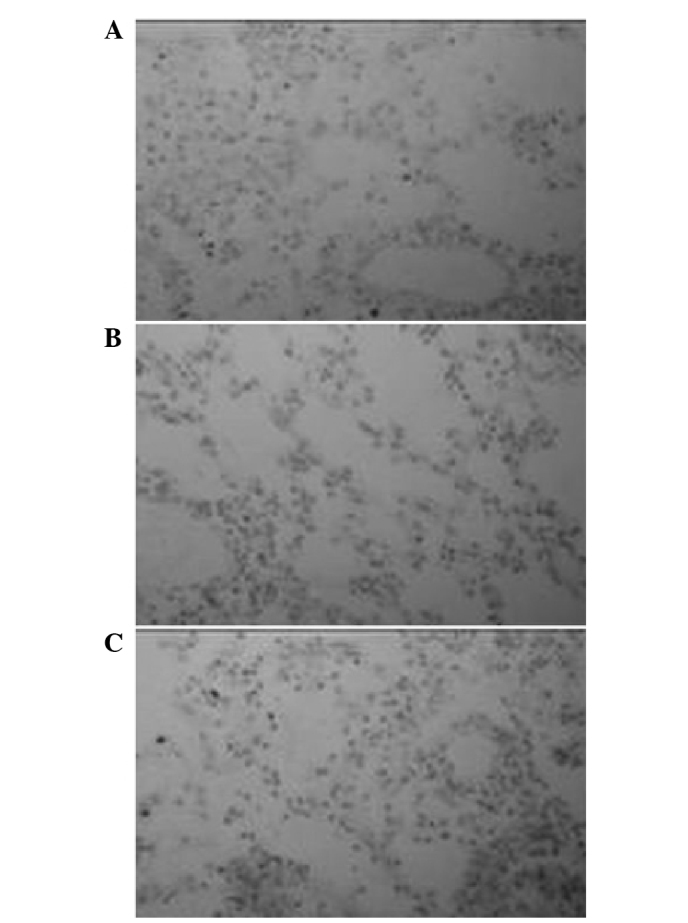
Xuebijing injection promoted the protein expression of the anti-inflammatory cytokine interleukin 10. Immunohistochemically stained tissue in the (A) treatment, (B) control and (C) model groups. Magnification, ×400.

The immune response products comprising the proinflammatory cytokine IL-6 and the anti-inflammatory cytokine IL-10, which are mainly located in the cytoplasm were examined by immunohistochemistry. Positively immunoreactive products with DAB staining were observed as brown-yellow granules. Examples of staining in each of the groups are shown in Figs. 3 and 4. By the χ^2^ test, the expression level of IL-6 protein in the TG was lower than that in the MG (P<0.05) and higher than that in the CG (P≥0.05), and the expression levels of the proinflammatory cytokine IL-6 and anti-inflammatory cytokine IL-10 in the TG were higher than those in the MG (P<0.05) and CG (P≥0.05), as shown in Tables I and II.

**Figure 5:**
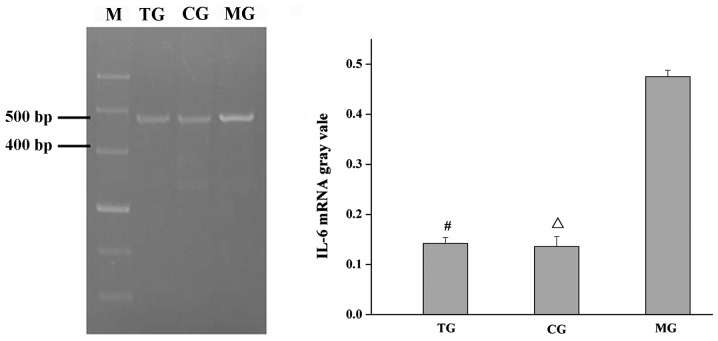
Xuebijing injection inhibited the mRNA expression of the pro-inflammatory cytokine IL-6. (A) mRNA expression of IL-6. Lane M is a 100-bp DNA ladder. (B) IL-6 mRNA gray vale. ^#,Δ^P<0.05 compared with MG. TG, treatment group; CG, control group; MG, model group; IL-6, interleukin 6.

**Figure 6:**
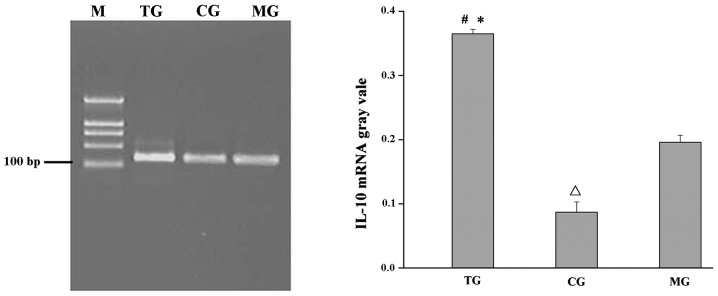
Xuebijing injection promoted the mRNA expression of the anti-inflammatory cytokine IL-10. (A) mRNA expression of IL-10. Lane M is a 100-bp DNA ladder. (B) IL-10 mRNA gray vale. ^#,Δ^P<0.05 compared with MG and ^*^P<0.05, TG compared with CG. TG, treatment group; CG, control group; MG, model group; IL-10, interleukin 10.

RT-PCR was used to further clarify the effect of Xuebijing injection on IL-6 and IL-10 protein expression levels in the lung tissue of rabbits with ALI induced by oleic acid. The results of the RT-PCR were highly consistent with the results of the immunohistochemistry, as shown in Figs. 5 and 6. Subsequent to the administration of the Xuebijing injection, the expression level of IL-6 mRNA clearly decreased, while the expression level of IL-10 MRNA further increased.

## Discussion

The objective of this study was to investigate the possible mechanisms by which Xuebijing injection exerts its effects in the treatment of ALI. The results showed that the improvement of respiratory function in ALI by Xuebijing injection may be associated with the inhibition of the mRNA and protein expression of the proinflammatory cytokine IL-6, and the enhancement of the mRNA and protein expression of the anti-inflammatory cytokine IL-10.

First, a classic model of ALI induced by oleic acid was established, and the pathophysiological changes and the response process of the oleic acid-induced ALI were close to clinical. The key to replicating the model of ALI was to accurately inject a specific amount of oleic acid. Oleic acid was injected into the ear vein of rabbits slowly and uniformly (0.1 ml/per min). The method was simple, with low trauma and the effect was significant. One hour subsequent to oleic acid being injected into the rabbits, the breathing of the rabbits significantly quickened, cyanosis of the lips was observed, inspiratory and expiratory crackles were clearly audible in the anterior right lung field, and the most typical phenomenon was that pink foamy liquid poured from the intubation.

Secondly, PaO_2_ and PaCO_2_, which are used to evaluate respiratory function, were measured. Blood gas analysis showed that the PaO_2_ and PaCO_2_ of rabbits in the MG were lower than those in the CG (Fig. 1), and the difference was statistically significant. This suggested that oleic acid impaired the lung and its effects were similar to the clinical changes associated with ALI, including progressive dyspnea and hypoxemia that are difficult to correct. The PaO_2_ and PaCO_2_ of rabbits in the TG were higher than those in the MG (Fig. 1), and were lower than those in the CG, although not statistically significantly, which implied that Xuebijing injection alleviated lung injury and improve respiratory function.

Thirdly, MPO activity, a marker of neutrophil activation, was detected. Activation and migration of neutrophils are regarded key events in the progression of ALI. Neutrophils are the first cells to be recruited to the sites of injury or infection (23,24) once an inflammatory response is initiated. An appropriate amount of neutrophils has a potent antimicrobial effect; however, an excessive amount of neutrophils can damage host tissues. MPO activity is used to quantify the amount of neutrophil infiltration. The present study demonstrated that MPO activity in the MG was higher than that in the TG and CG, and the differences were statistically significant (Fig. 2), which identified that numerous neutrophils were activated by oleic acid in the pulmonary circulation of the MG rabbits, and activated neutrophils led to ALI and respiratory dysfunction (decreased PaO_2_ and PaCO_2_; Fig. 1). Xuebijing injection effectively reduced the number of activated neutrophils (decreased MPO activity; Fig. 2), and the respiratory function improved (increased PaO_2_ and PaCO_2_; Fig. 1).

Fourthly, the protein and mRNA expression levels of the proinflammatory cytokine IL-6 and the anti-inflammatory cytokine IL-10 in the lung tissue were detected by immunohistochemistry and the RT-PCR technique. The results demonstrated that the protein and mRNA expression levels of IL-6 and IL-10 in the lung tissue of the MG were increased. Oleic acid exerted an effect on the body; inflammatory cells and immune cells were in a highly activated state, large quantities of IL-6 and IL-10 were released into the blood, the rabbits showed rapid shallow breathing, the PaCO_2_ decreased, the number of activated white blood cells increased (increased MPO activity) and other typical clinical signs of ALI were observed. However, Xuebijing injection is able to regulate the expression of inflammatory cytokines (25–27). Xuebijing injection reduced the number of neutrophils (reduced MPO activity; Fig. 2), reduced the expression of the proinflammatory cytokine IL-6 (Figs. 3 and 5, Table I), and increased the expression of the anti-inflammatory cytokine IL-10 (Figs. 4 and 6, Table II) at the mRNA and protein levels. The inflammation was localized.

In the oleic-induced pathogenesis of ALI, oleic acid, a harmful factor, invaded the pulmonary circulation and neutrophils were activated. Activated neutrophils underwent chemotaxis, aggregation and release reaction, and the proinflammatory cytokine IL-6 was over-secreted, which resulted in imbalanced metabolic pathways and tissue damage. To prevent severe damage, the anti-inflammatory cytokine IL-10 was released and an vigorous, uncontrolled inflammatory response occurred, which led to lung tissue damage and function injury. Xuebijing injection effectively reduced neutrophil adhesion, aggregation and release reaction (MPO activity weakened), reduced the levels of the proinflammatory cytokine IL-6 and increased the levels of the anti-inflammatory cytokine IL-10. Thus, inflammation was effectively controlled and respiratory function improved.

## Figures and Tables

**Table I:** Comparison of the protein expression of interleukin 6 in the lung tissue of the rabbits in the three groups (n=20).

	Interleukin 6				
	
Group	−	+	++	+++	P-value
TG	8	8	3	1	<0.05^a^
MG	2	4	9	5	<0.05^b^
CG	7	9	4	0	≥0.05^c^

These experiments were conducted as independent triplicates.

a TG vs. MG;

b CG vs. MG;

c TG vs. CG.

TG, treatment group; CG, control group; MG, model group.

**Table II:** Comparison of the protein expression of interleukin 10 in the lung tissue of rabbits in the three groups (n=20).

	Interleukin 6				
	
Group	−	+	++	+++	P-value
TG	0	4	7	9	<0.05^a^
MG	1	2	6	11	<0.05^b^
CG	9	5	4	2	≥0.05^c^

These experiments were were conducted as independent triplicates.

a TG vs. MG;

b CG vs. MG;

c TG vs. CG.

TG, treatment group; CG, control group; MG, model group.

## Abbreviations

IL-6: interleukin 6
IL-10: interleukin 10
TG: treatment group
MG: model group
CG: control group
MODS: multiple organ dysfunction syndrome
PaO_2_: arterial partial pressure of oxygen
PaCO_2_: arterial partial pressure of carbon dioxide
MPO: myeloperoxidase
ALI: acute lung injury
TGF-β: transforming growth factor-β
SIRS: systemic inflammatory response syndrome
ELISA: enzyme-linked immunosorbent assay
RT-PCR: reverse transcription-polymerase chain reaction
DAB: 3,3′-diaminobenzidine
